# Modeling and Exploring Stillbirth Risks in Northern Pakistan

**DOI:** 10.3390/healthcare13121436

**Published:** 2025-06-16

**Authors:** Muhammad Asif, Maryam Khan, Saba Tariq

**Affiliations:** 1 Department of Mathematics and Statistics, College of Science, Imam Mohammad Ibn Saud Islamic University (IMSIU), Riyadh 11623, Saudi Arabia; 2 Department of Statistics, University of Malakand, Chakdara 18800, Lower Dir, Pakistan; 3 Department of Metabolism and Systems Science, College of Medicine and Health, University of Birmingham, Birmingham B15 2TT, UK; s.tariq@bham.ac.uk

**Keywords:** maternal, public health, epidemiology, biostatistics

## Abstract

**Background:** The World Health Organization (WHO) defines stillbirth as the loss of a fetus after 28 weeks of gestation. Annually, approximately 2 million stillbirths occur worldwide. Projections indicate that by 2030, this figure could rise to nearly 15.9 million, with half of these stillbirths expected to take place in Sub-Saharan Africa. In the global literature, causes include placental complications, birth defects, and maternal health issues, though often the cause is unknown. Stillbirths have significant emotional and financial impacts on families. **Methods:** The process involves using chi-square tests to identify candidate covariates for model building. The relative risk (RR) measures the association between variables using the sample data of 1435 mothers collected retrospectively. Since these tests are independent, covariates might be interrelated. The unadjusted RR from the bivariate analysis is then refined using stepwise logistic regression, guided by the Akaike Information Criterion (AIC), to select the best subset of covariates among the candidate variables. The logistic model’s regression coefficients provide the adjusted RR (aRR), indicating the strength of the association between a factor and stillbirth. **Results:** The model fit results reveal that heavy bleeding in the second or third trimester increases stillbirth risk by 4.69 times. Other factors, such as water breaking early in the third trimester (aRR = 3.22), severe back pain (aRR = 2.61), and conditions like anemia (aRR = 2.45) and malaria (aRR = 2.74), also heightened the risk. Further, mothers with a history of hypertension faced a 3.89-times-greater risk, while multifetal pregnancies increased risk by over 6 times. Conversely, proper mental and physical relaxation could reduce stillbirth risk by over 60%. Additionally, mothers aged 20 to 35 had a 40% lower risk than younger or older mothers. **Conclusions:** This research study identifies the significant predictors for forecasting stillbirth in pregnant women, and the results could help in the development of health monitoring strategies during pregnancy to reduce stillbirth risks. The research findings further support the importance of targeted interventions for high-risk groups.

## 1. Introduction

The definition of “stillbirth” varies between countries. For instance, in the US and England, stillbirth is considered the loss of a fetus after 24 weeks of gestation [1,2]. The World Health Organization (WHO) defines stillbirth as the death of a fetus at or after 28 weeks of pregnancy. In Pakistan, healthcare providers classify stillbirth as per the WHO definition. Approximately two million stillbirths occur annually worldwide [3]. According to the WHO global projection, if the current trend continues, nearly 15.9 million stillbirths could happen by 2030, with almost half of these stillbirths being expected to occur in Sub-Saharan Africa. Though, in many cases, the exact cause of stillbirth remains unknown, stillbirth can occur due to various reasons, including complications with the placenta, birth defects, maternal health issues, or even environmental conditions such as extreme heat and air pollution [4,5,6]. In medical studies, to a certain extent, the exact cause of stillbirths can be determined by complete diagnostic autopsy (CDA) or minimally invasive tissue sampling (MITS) methods postpartum. However, postpartum evaluation is usually not feasible due to limited data coverage, complex implementation, and resource constraints, especially in the case of the CDA method, which is globally recommended [7]. Experiencing stillbirth is a deeply emotional and challenging event for families, often accompanied by psychological and financial repercussions [8,9,10].

According to the UNICEF’s 2021 annual report, globally, the estimated average rate of stillbirth in that year was 13.9 per 1000 births; however, this is perhaps an underestimation because of the issue of underreporting [11]. As part of the Every Newborn Action Plan (ENAP), the WHO’s global target aims to reduce the stillbirth rate to less than 12 per 1000 births by 2030. In this regard, worldwide, a considerable reduction in the stillbirth rate has been observed since 2000 [12]. For instance, Tanzania has effectively reduced its stillbirth rate by implementing a specialized program, namely the Safer Births Bundle of Care (SBBC) program [13]. However, the issue remains serious in low- and middle-income countries, such as Pakistan. According to the 2023 progress report on maternal and newborn health, Pakistan continues to face significant challenges in reducing stillbirth rates [14]. Shakeel et al. [8] reported that Pakistan has one of the highest stillbirth rates in South Asia, with a significant increase in the stillbirth rate observed between 2012 and 2017. Various studies report an alarming situation in developing countries like Pakistan, estimating up to 70 stillbirths per 1000 births on average [5,15,16,17,18].

Regarding the causes, several studies have reported that fetal growth restriction (FGR) is a significant cause of stillbirth [18,19]. FGR occurs when a baby does not grow at the expected rate in the womb due to placental insufficiency—a condition where the placenta fails to supply adequate oxygen and nutrients to the fetus [20]. Likewise, pre-existing maternal health conditions like hypertension, diabetes, and infectious diseases are important factors that significantly increase the likelihood of stillbirths [5,21,22,23,24,25,26]. Further, some studies have reported that mothers over 35 years of age have a higher risk of stillbirths [25,27,28,29,30,31,32]. In contrast, DeMarco, Twynstra [33] and Engmann, Garces [34] reported that women at younger ages are at greater risk of stillbirth. Researchers have also reported pregnancy complications [35] (e.g., heavy bleeding), lifestyle factors [36] (e.g., food nutrition, smoking, alcohol, or drugs, etc.), and socio-economic factors [37,38] contribute to the higher risk of stillbirths.

This study identifies and investigates stillbirth’s associated risk factors using Generalized Linear Model (GLM). Specifically, logistic regression with a binary response variable, stillbirth (yes/no), was used to determine risk factors associated with stillbirth using data from northern Pakistan and enhance the consistency and validity of previous results. Further, to our knowledge, previously, no such study has been conducted in the specified region using advanced statistical models. Our recommendations will help in the development of useful public health and clinical strategies to control stillbirths and decrease the morbidity and mortality associated with stillbirths. In the Section 2, we describe the methods and materials used, for example, the data sources and statistical methods employed for data analysis. The results are provided and discussed in Section 3, the study limitations are acknowledged in Section 4, and concluding remarks are provided in Section 5.

## 2. Methods and Materials

### 2.1. Study Area and Ethical Statement

This study was conducted in the Swat District of Pakistan. Pakistan is a low-income South Asian country, while the Swat District is the 15th largest district (by population) of the Khyber Pakhtunkhwa province, consisting of 62 Union Councils. According to the 2017 census figures, the population of District Swat was 2,309,570, consisting of 1,136,544 female individuals.

Ethical approval for the study was granted by the Advanced Study and Research Board (ASRB) of Malakand University. Consent was not required as the data analyzed was anonymized; despite this, from each participant, verbal consent was obtained via phone call after explaining that the anonymized data would be used solely for research purposes. Each participant was informed that they could skip any question and could end the phone call if they were not willing to participate in the interview. Subsequently, a structured questionnaire was completed by the participants through a phone call in May 2020; phone calling was necessary as there was a lockdown in the region due to the COVID-19 pandemic.

### 2.2. Prevalence and Sampling

This study involved a random sample of 1435 mothers who gave birth in the Swat District, Pakistan, between January and August 2019. The data was collected retrospectively in the month of May 2020 via telephone calls. Within this cohort, 67 cases (4.7%) resulted in stillbirths. A simple random sampling technique from the sampling framework provided by the district Swat headquarters hospital (DHQ Swat) was used.

### 2.3. Statistical Methods

This research project employed logistic regression within the Generalized Linear Model (GLM) framework. First, we conducted a series of independent chi-square tests to assess associations in order to identify significant (at 5% level) candidate covariates for model building. Further, we used relative risk (RR) measures to quantify the association between the categorical variables. The RR is the ratio of the probability of the event (e.g., stillbirth) in the exposed group (e.g., hypertension) to the probability of the event in the unexposed group. Since these tests are performed independently, the covariates will likely be interrelated and provide similar information. Further, the relative risk value obtained from the bivariate analysis is considered unadjusted for the effect of other variables on the stillbirth.

Therefore, in the next step, we performed stepwise logistic regression in both backwards and forward directions to obtain the best independent subset of covariates for inclusion in the final model. The AIC (Akaike Information Criterion) was used to determine whether the model should include the covariate. Stepwise regression using the Akaike Information Criterion (AIC) offers several advantages in model selection. Firstly, it balances goodness-of-fit and complexity—the AIC penalizes excessive model complexity, helping to avoid overfitting while still selecting a model that fits the data well. Secondly, stepwise regression systematically adds or removes variables based on the AIC, especially variables that are significantly correlated, hence reducing the problem of multicollinearity. Thirdly, it improves model interpretability by eliminating unnecessary predictors, producing a simpler model that is easier to interpret. Finally, this procedure is efficient for large datasets, compared to exhaustive search methods; stepwise regression with the AIC is computationally efficient [39,40,41].

Additionally, the exponential of the estimated regression coefficient from the logistic model provides the adjusted relative risk (aRR). Thus, the aRR determined from the model can be considered as the adjusted strength of association between the covariate (e.g., hypertension) and the response variable (stillbirth). All of the analyses were performed in IBM SPSS version 25 and R version 4.5.0 [42].

## 3. Results and Discussions

### 3.1. Descriptives

The age of the participating mothers spanned from 16 to 45 years, with an average age of 27.7 years and a standard deviation of 6.8 years. Approximately 66% or 948 mothers were aged between 20 and 35 years. A significant majority (1233 mothers or 85.9%) resided in rural areas, and a large proportion (1161 mothers or 81.0%) were illiterate. Nearly all participants, 1421 mothers (99.2%), were housewives. Approximately 35% of the respondents were financially unstable and struggling to fulfill their basic needs. Among the pregnancies, there were 54 instances (3.8%) of twins. Moreover, approximately 15% of mothers underwent cesarean deliveries. This comprehensive dataset underscores the complex interplay of maternal health, socio-economic factors, and family dynamics in influencing pregnancy outcomes in the Swat District. Table 1 and Table 2 provide data descriptions in the form of count summaries and percentages for the categorical variables.

### 3.2. Bivariate Analysis

A bivariate analysis was conducted to investigate the association of stillbirth with various risk factors using chi-square tests at a 5% significance level. The unadjusted relative risk (RR) measures indicated that several variables were significantly associated with stillbirth, with a *p*-value of less than 0.05. The unadjusted measure for RR means that the strength and significance of the associations have no adjustment for the other significant variables that affect stillbirth. Table 3 presents case summaries, the unadjusted RR measures, and test results regarding the associations of the twenty variables that were found to be statistically significant during the analysis.

Nine variables relevant to pregnancy complications or health issues during pregnancy were found to be significant. One of these is severe bleeding during the second/third trimester, with 20.7% stillbirths in the exposed group, and the value of unadjusted relative risk (RR) indicates that mothers with symptoms of severe bleeding are 5.75 times more likely to experience stillbirths as compared to mothers with no such symptoms. Likewise, in the sample data, 664 mothers reported back pain, with 55 (or 8.3%) stillbirths occurring. The relative risk measure was calculated as 5.19, indicating that mothers with the issue of severe back pain have a 5.19-times-higher risk.

Other variables identified as significant during the bivariate analysis and relevant to the pregnancy complications were anemia (9.0%, RR = 3.21), eclampsia (25%, RR = 5.68), continuous headache (7.8%, RR = 2.51), vaginal infection (7.3%, RR = 2.28), and water breaks in the early stage of the third trimester (9.7%, RR = 2.55). Interestingly, mothers with a feeling of nausea are approximately 40% less likely to have a stillbirth as compared to mothers with no sense of nausea. Finally, if a mother has malaria (10.2%, RR = 2.61) during pregnancy, she will have a 2.61-times-higher risk of experiencing stillbirth.

Regarding the variables relevant to maternal health history, our bivariate analysis results show that 10.5 per cent of stillbirths occurred among hypertensive pregnant women, with the value of relative risk indicating that hypertensive pregnant women are 3.5 times more likely to have a stillbirth. Likewise, diabetes (12.5%, RR = 2.78), cardiovascular disease (11.8%, RR = 3.81), self-reported depression (8.0%, RR = 2.35), and miscarriage history (6.9%, RR = 1.73) were found to be statistically significant in the bivariate analysis using the chi-square test.

Moreover, maternal age was found to be a significant factor, with the highest incidence of stillbirths (8.8%, RR = 2.4) occurring among mothers over 35 years old. This was followed by very young mothers (under 20 years old), who had a stillbirth rate of 5.5% (RR = 1.52). The results of the chi-square test assessing the association between the response variable (stillbirth) and binary variable age (takes the value 1 if the mother was aged between 20 and 35 years old and 0 otherwise) show that the risk may be reduced by half if the mother’s age is between 20 and 35 years old (*p* = 0.004).

In addition to these factors, the crude bivariable analysis found other associations with stillbirth. For example, financial instability (7.1%, RR = 2.09) and regular walking or recommended exercises (2.8%, RR = 0.48) were identified as associations. These results show that the risk may be reduced by more than half if a mother performs recommended physical exercises. Further, the results also show that relaxation significantly reduces the risk of stillbirth. For example, mothers who do not engage in strenuous activities at home (such as hard cleaning, loading, etc.) or perform just effortless/light work (e.g., cooking, etc.) at home are 55% less likely to have stillbirth.

Interestingly, some variables were statistically nonsignificant in the bivariate analysis. For example, the number of antenatal care visits was statistically insignificant (*p* = 0.08). This result is contrary to what was reported in [43]. This does not mean that antenatal visits are unimportant; rather, it may be due to their positive relationship with poor health conditions. Mothers with severe symptoms and poor health conditions are expected to visit health centers more frequently. As a result, to a certain extent, this cancels out the negative effect of antenatal visits with the positive effect of health conditions on the probability of stillbirth. Furthermore, we do not have sufficient evidence to conclude that a mother’s place of residence (urban or rural) has a significant effect on stillbirth. Additionally, our analysis indicates that the likelihood of stillbirth is the same for both male and female fetuses. In contrast, Sheiner, Levy [44] reported that mothers carrying male fetuses have a higher risk of pregnancy complications that may lead towards stillbirth.

### 3.3. Model Fit Results

Our bivariate analysis identified 20 statistically significant variables affecting the binary response variable, stillbirth. However, many of these variables are highly correlated and provide similar information or, in statistical terminology, have a confounding effect. For example, hypertension, depression, and headaches could be associated factors [45,46]. Additionally, the relative risk (RR) was not adjusted to account for the effects of other variables. Moreover, a series of independent statistical tests may increase the Type I error. One possible way of tackling the issue is multiple comparison test adjustment, but, in general, this is less efficient as compared to statistical modeling. Therefore, we modeled stillbirth to identify the best subset of covariates and adjust the RR for each associated variable, considering the effects of other important covariates.

**Figure 1:**
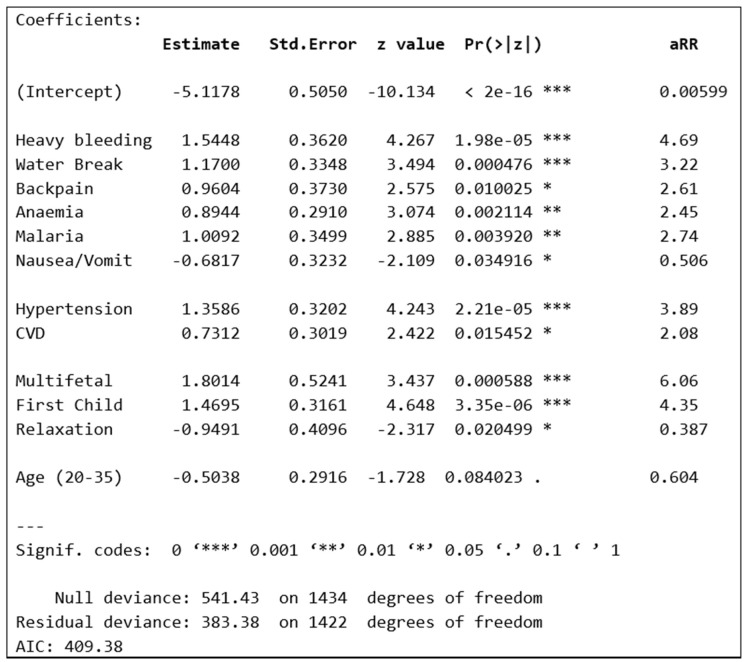
The best-fitted model results using purposely written R [42] programming codes.

All 20 candidate covariates were included in a binary logistic regression model, and stepwise selection in backward and forward directions was performed using the Akaike Information Criterion (AIC) [47]. This modeling approach was employed to estimate the effects of various behavioral, health, and socio-demographic characteristics, aiming to identify independent potential risk factors for stillbirth. The results in Figure 1 include relative risk (aRR), and *p*-values. Moreover, all model diagnostics were thoroughly checked, and no issues were found in terms of model adequacy or inferential statistics.

The adjusted relative risk (aRR) value obtained from the fitted model indicated that mothers with heavy bleeding during the second or third trimester have an increased risk of stillbirth by 4.69 times compared to those with no such symptoms, provided that other covariates in the model remained constant. A leading cause of heavy bleeding during late pregnancy is placental abruption followed by placenta previa and an incompetent cervix [48,49]. Placental abruption is when the placenta detaches from the uterus before delivery, which can lead to significant bleeding and is a major risk factor for stillbirth [50].

Likewise, water breaking during the early stage of the third trimester has a more than threefold-increased risk of stillbirth, as the aRR value is equal to 3.22. Further, the results from the fitted model show that the stillbirth likelihood may be increased by 2.61 times if the mother had severe back pain issues during pregnancy. Further, women with anemia are at a 2.45-times-higher risk, while those who have experienced malaria during pregnancy have a 2.74-times-increased risk of stillbirth. Moreover, the model fit results also indicate that, by keeping other factors constant, the risk may be reduced by approximately half among mothers with symptoms of nausea/vomiting.

Furthermore, the model fit results also highlighted that some health- or medical history-related covariates are significantly associated with stillbirth. For instance, as shown in Figure 1, mothers with a history of hypertension indicate that the likelihood of stillbirth is higher by 3.89 times as compared to non-hypertensive mothers. Likewise, cardiovascular disease (CVD), with an aRR equal to 2.08, affects the risk of stillbirth, increasing it by more than double.

The results presented above are in line with the literature; for instance, previously reported health conditions significantly associated with stillbirth include hypertension, diabetes, and infectious diseases [5,21,22,23,24,25,26,51]. The researchers highlighted that the combination of impaired placental function, increased risk of complications, and potentially fatal growth issues contributes to an elevated risk of stillbirth in hypertensive mothers [52,53,54,55,56]. Moreover, the model fit results indicate that the risk of stillbirth may increase by more than sixfold among mothers with multifetal pregnancies. Likewise, the risk increases more than fourfold among mothers with their first child (see Figure 1). Further, proper mental and physical relaxation of the mother during pregnancy reduces the stillbirth risk by more than 60% (=100 × (1 − 0.387)).

Finally, keeping other factors fixed, the risk of stillbirth is reduced by 40% if the mother’s age during pregnancy is between 20 and 35 years old. This result is in line with what has been reported in the global literature: the likelihood of stillbirth among adolescents (aged less than 20 years) or adults aged more than 35 years is significantly higher. The literature highlights the causes associated with major congenital anomalies and maternal disorders. For example, placentas in older mothers often do not function as effectively, and this can lead to insufficient nutrient and oxygen supply to the fetus, which is crucial for fetal growth and development [25,27,28,29,30,31,32,33,57].

These results underscore the importance of monitoring and managing health conditions during pregnancy to mitigate the risk of stillbirth. Further research is needed to explore the underlying mechanisms and develop targeted interventions for these high-risk groups.

### 3.4. Policy Discussion and Implications

The results of this study have several practical implications for maternal healthcare and public health strategies aimed at reducing stillbirth rates in Pakistan and elsewhere in the world. Firstly, it is important to enhance monitoring and maternal care. For example, healthcare providers should closely and regularly monitor pregnant women exhibiting symptoms such as heavy bleeding, severe back pain, or early water breaking. Early intervention can be crucial in managing risks associated with conditions like placental abruption and placenta previa. Secondly, attention should be given to education and awareness. For example, educating expectant mothers about the signs of complications and the importance of seeking immediate medical attention may help reduce risks. This includes raising awareness about the dangers associated with heavy bleeding and other warning signs.

Thirdly, the management of pre-existing conditions, such as hypertension and cardiovascular disease, is essential. For instance, women with a history of hypertension or cardiovascular disease should receive specialized care and monitoring throughout their pregnancy to mitigate the increased risk of stillbirth. Additionally, given the increased risks associated with anemia and malaria, healthcare systems should prioritize screening and treatment for these conditions, especially in regions where these conditions are prevalent. Moreover, the findings suggest that mental and physical relaxation can significantly reduce stillbirth risk. Implementing programs that support maternal mental health, such as counseling and stress relief workshops, could be beneficial.

The results also highlighted that targeted prenatal care for younger mothers (under 20) and older mothers (over 35) is essential, as these groups face higher risks. Tailoring healthcare services to address the unique needs of these age groups can improve outcomes. Given the heightened risk for mothers with multifetal pregnancies, specialized prenatal care and monitoring should be standard practice for these patients. Continued research into the underlying mechanisms of these risk factors is necessary to develop effective interventions. Policymakers should consider these findings when designing maternal health programs and policies. By implementing these strategies, healthcare providers can better manage risks associated with stillbirth and improve maternal and fetal health outcomes. Apart from programs specifically targeting the issue of stillbirth, other programs like the Pakistan Poverty Alleviation Fund (PPAF) help improve financial conditions, which indirectly help improve health conditions [58].

## 4. Limitations

There are some limitations to this study. Firstly, due to the cultural norms in our sample data, no mother was a smoker, alcohol consumer, or drug addict, and therefore, it was not possible to determine the effect of such covariates. Further, in our sample data, only women who visited the healthcare center or hospital at least once were included. Therefore, to some extent, there may be a problem of underestimation as cases may be underreported in remote areas where women have no access to health facilities. Further, the data is representative of the northern area of Pakistan, and therefore, the results may not be generalizable to the other parts of Pakistan, especially the southern part of Pakistan. This is due to the high heterogeneity in the population of Pakistan.

Further, as we used cross-sectional data, a time series model could not be applied to determine seasonal effects. For example, the authors of [59] utilized time series data to investigate the effect season has on deaths. Further, the results may not be considered as derived from a cause–effect analysis; rather, they should be considered as statistical associations. Moreover, to some extent, sample bias may exist, since the data was collected via telephone calls. As a result, some participants included in the sample could not be reached due to changes in their mobile or telephone numbers, or because they had passed away.

## 5. Conclusions

The analysis identified 20 significant variables affecting stillbirth, but many were correlated, leading to potential confounding effects. A binary logistic regression model was employed to refine these variables and adjust the relative risk for each. The key findings include the fact that heavy bleeding in the second or third trimester increases stillbirth risk by 4.69 times. Water breaking early in the third trimester raises the risk by 3.22 times. Severe back pain during pregnancy correlates with a 2.61-times-higher risk. Anemia or malaria during pregnancy increases the risk by more than double. In addition, a history of hypertension raises the risk by 3.89 times, while cardiovascular disease doubles the risk. Multifetal pregnancies increase the risk by over six times. Proper mental and physical relaxation can reduce the risk by more than 60%. Further, mothers aged 20 to 35 have an approximately 40% lower risk compared to adolescents (less than 20 years) or adults over 35 years old.

These results highlight the critical need for monitoring and managing health conditions during pregnancy to mitigate stillbirth risk. This study underscores the importance of targeted maternal healthcare strategies to reduce stillbirth rates. Key recommendations include enhanced monitoring, including regular check-ups for pregnant women showing symptoms like heavy bleeding or severe back pain and informing expectant mothers about complications and the importance of seeking timely medical help; management of pre-existing conditions, like specialized care for women with hypertension or cardiovascular issues; programs to promote mental and physical relaxation during pregnancy; and focused services for younger and older mothers and those with multifetal pregnancies. Continued research is essential in order to understand these risk factors and their mechanisms and develop effective interventions. Policymakers should integrate these findings into maternal health programs to improve outcomes for mothers and infants.

## Figures and Tables

**Table 1:** Count summaries of the sample data.

Variable	Cases (Percentage)
Mother (Illiterate)	1161 (81.0%)
Mother (Housewife)	1421 (99.2%)
Financially Unstable	493 (34.3%)
Residence (Rural)	1230 (85.9%)

**Table 2:** Count summaries for medical history and pregnancy complications.

Variable	Cases	Variable	Cases
Anemia	431 (30.1%)	Heavy bleeding	92 (6.4%)
Hypertension	324 (22.6%)	Premature births	71 (4.6%)
Depression	301 (21.0%)	Abnormal fetal position	44 (3.1%)
Cardiovascular disease	263 (18.3%)	Premature (water breaks)	216 (15.1%)
Kidney problems	84 (5.9%)	Eclampsia	16 (1.1%)
asthma	58 (4.0%)	Vaginal infections	520 (36.3%)
diabetes	32 (2.2%)	Twins	54 (3.8%)
Headaches	487 (34.0%)	Back pain	664 (46.3)
Malaria	177 (12.3%)	Nausea and vomiting	1054 (73.5%)

**Table 3:** Results derived from testing a series of independent chi-square tests for the association of stillbirth and different factors.

		Factor	Stillbirth Cases (*n*)				Statistical Tests	
			Non-Exposed		Exposed		Un-Adj	*p*-Value
	S.No		*n*	Percent	*n*	Percent	RR	LR
Pregnancy Complications	1.	Heavy bleeding	48	3.6%	19	20.7%	5.75	0.000
	2.	Anemia	28	2.8%	39	9.0%	3.46	0.000
	3.	Eclampsia	63	4.4%	4	25.0%	5.68	0.005
	4.	Headaches	29	3.1%	38	7.8%	2.52	0.000
	5.	Backbone pain	12	1.6%	55	8.3%	5.19	0.000
	6.	Vaginal infection	29	3.2%	38	7.3%	2.28	0.001
	7.	Water breaks	46	3.8%	21	9.7%	2.55	0.001
	8.	Nausea/vomiting	25	6.6%	42	4.0%	0.61	0.047
	9.	Malaria	49	3.9%	18	10.2%	2.62	0.001
Health history	10.	Hypertension	33	3.0%	34	10.5%	3.50	0.000
	11.	Diabetes	63	4.5%	4	12.5%	2.78	0.075
	12.	Depression	43	3.8%	24	8.0%	2.11	0.004
	13.	CVD	36	3.1%	31	11.8%	3.81	0.000
Other factors	14.	Multiple pregnancies	59	4.3%	8	14.5%	3.37	0.004
	15.	Miscarriage history	44	4.0%	23	6.9%	1.73	0.032
	16.	Age (20 to 35)	34	7.0%	33	3.5%	0.5	0.004
	17.	Recommended exercise	53	5.8%	14	2.8%	0.48	0.009
	18.	Relaxation	57	5.5%	10	2.5%	0.45	0.013
	19.	Financially unstable	32	3.4%	35	7.1%	2.09	0.002
	20.	First child	36	3.5%	31	7.4%	2.11	0.002

## Data Availability

The data presented in this study are available on request from the corresponding author.
